# Synthesis of self-curable polysulfone containing pendant benzoxazine units via CuAAC click chemistry

**DOI:** 10.1080/15685551.2016.1257379

**Published:** 2016-11-21

**Authors:** Cemil Dizman, Cagatay Altinkok, Mehmet Atilla Tasdelen

**Affiliations:** ^a^ Institute of Chemical Technology, TUBITAK Marmara Research Center, Kocaeli, Turkey; ^b^ Faculty of Engineering, Department of Polymer Engineering, Yalova University, Yalova, Turkey

**Keywords:** Benzoxazine, click chemistry, cross-linking, polybenzoxazine, polysulfone

## Abstract

Synthesis, characterization, and properties of new thermally curable polysulfone containing benzoxazine moieties in the side chain were investigated. First, chloromethylation and subsequent azidation processes were performed to form polysulfone containing pendant clickable azide groups. Independently, antagonist 3,4-dihydro-3-(prop-2-ynyl)-2H-benzoxazine was prepared by using paraformaldehyde, phenol and propargylamine. The following copper(I) catalyzed azide-alkyne cycloaddition click reaction was applied to obtain self-curable polysulfone with pendant benzoxazine units. The polymer and intermediates at various stages were characterized by ^1^H-NMR, ^13^C-NMR and FT-IR spectroscopies. The thermal properties and curing behavior of final polymer were investigated by differential scanning calorimetry and thermal gravimetric analysis. Compared to the neat polysulfone, the obtained polymers exhibited thermally more stable polymers.

## 1. Introduction

The synthesis of novel materials for high-tech applications such as electronics, medical, automotive, and aerospace has received great deal in recent years. Researchers have prepared many different polymers displaying improved chemical and physical properties by reacting polymers with the functional groups on them or by modification of a polymer with a reactive group that can be used for addition of new polymeric units. As a commercially important polymer, polysulfones (PSU) are a class of engineering polymers with extra-ordinary features comprising thermal stability, durability in harsh conditions, oxidation, pH and temperature resistance, excellent mechanical strength, ease of processability and good film properties.[1–3] They are widely utilized in many different applications such as, nano or micro filtration membranes, coatings, composites, microelectronic devices, thin-ﬁlm technology, and biomaterials.[4] Polysulfones can be converted to novel polymers having enhanced physical and chemical properties that fulfills the needs of market. New functional groups or polymeric units can be added by end chain or side chain (graft) chemical modification. In our previous works we studied the end chain modification of polysulfone oligomers and their usage in crosslinking network and nanocomposite formations.[5–11] Other researches also applied different side chain modifications in order to obtain grafting polysulfones with different functional groups.[12–15]

Benzoxazines are a novel class of thermosetting compounds extensively studied in polymer science due to their self-curable properties upon heating; i.e. there is no need for an additional curing agent.[16–20] They were simply blended with or incorporated into various polymers such as polystyrenes, polyurethanes, and others.[21–25] By opening of the benzoxazine ring at elevated temperatures, thermally and mechanically more durable polymeric networks were formed.[26–30] The synthesis and curing behavior of benzoxazine chemistry and its potential applications were previously reviewed in the literature.[16,17,19,29] There were limited studies for the combination of polysulfone with benzoxazine as either in the main chain or terminal groups. By simple heating of these polymers not only allowed to obtain new polymeric networks with enhanced properties such as high mechanical and thermal stabilities but also to get novel properties like self-healing property.[31–33]

Due to its simplicity, versatility, efficiency and speed, the copper catalyzed azide-cycloaddition (CuAAC) click reaction was employed for the synthesis of many organic,[34,35] inorganic and hybrid materials.[36–38] It has been extensively utilized for synthesis or modification of thermoplastic and thermoset polymers.[39–46] Several examples related to the application of the CuAAC click reaction for the modification of polysulfones were previously studied.[13,14,24,47–51] However, there was no report on the introduction of benzoxazine units at the side-chain position of polysulfones using chemistry reactions or other efficient organic reactions. In this study, self-curable polysulfone containing pendant benzoxazine units was prepared via CuAAC click chemistry strategy. The thermal properties and curing behavior of the obtained polymer were investigated by differential scanning calorimetry (DSC) and thermogravimetric analysis (TGA).

## 2. Experimental

### 2.1. Materials

Polysulfone, (PSU UDEL P-1700, *M*_n _= 30.000 g/mol) was obtained from Solvay Advanced Polymers and used without purification. Paraformaldehyde (Aldrich, 95%), tin(IV)chloride (SnCl_4_, Aldrich, 99%), and chlorotrimethylsilane ((CH_3_)_3_SiCl, Merck, 99%), chloroform (Aldrich, 99%), N,N-dimethylformamide (Merck, 99%), 1,4-dioxane (Aldrich, 99%), methanol (Aldrich, 99%), ethanol (technical grade), sodium azide (Merck, 99%), copper (I) bromide (Acros Organics, 98%), 2,2′-dipyridyl (Acros Organics, 99%) and propargylamine (Aldrich, 98%) were used without any further purifications.

### 2.2. Synthesis of 3,4-dihydro-3-(prop-2-ynyl)-2H-benzooxazine monomer (Pg–Bz)

The Pg***–***Bz was prepared according to published procedure [52]: phenol (36 mmol, 3.42 g), and propargyl amine (18 mmol, 1.00 g) were dissolved in 1,4-dioxane (30 mL) in a 50 ml three necked ﬂask and then paraformaldehyde (72 mmol, 2.18 g) was slowly added into the mixture. After 10 min later, the mixture warmed to 110 °C and was reﬂuxed this temperature for 12 h. The crude product was dissolved in diethyl ether and washed several times with 3 N sodium hydroxide solution and ﬁnally with distilled water. Then, the ether solution was dried over sodium sulfate anhydrous, followed by evaporation of solvent under vacuum to afford pale yellow viscous ﬂuid.

FT-IR (ATR, cm^−1^): 3290 (≡C–H), 2120 (C≡C), 1490 (Ar) and 1230 (C–O–C).

^1^H-NMR (CDCl_3_, ppm): *δ* = 7.4–6.8 (m, Ar, benzoxazine), 4.9 (s, N–CH_2_–O, benzoxazine), 4.1 (s, N–CH_2_–C, propargyl), 3.6 (s, N–CH_2_–C, benzoxazine), and 2.3 (≡C–H, propargyl).

^13^C-NMR (CDCl_3_, ppm): *δ* = 44.4 (N–C–C≡, propargyl) 51.3 (Ar–C–N, benzoxazine), 73.1 (C≡C, propargyl), 78.2 (O–C–N, benzoxazine), 78.8 (C≡C, propargyl), 113.2, 120.4, 126.7, 129.1, 130.2 and 153.9 (Ar, benzoxazine).

### 2.3. Chloromethylation of polysulfone (PSU–CH_2_–Cl)

PSU (0.5 mmol, 15.00 g) was completely dissolved in 750 mL of chloroform in a round-bottom flask under inert atmosphere at 50 °C. After that trichloromethylsilane (CH_3_)_3_SiCl (225 mmol, 28.50 mL) were added slowly, drop by drop, to the flask. Paraformaldehyde (225 mmol, 6.70 g) was poured into the reaction mixture in one portion. Finally, tin (IV) chloride (SnCl_4_) (2.25 mmol, 0.26 mL) was added slowly by a syringe and the mixture was stirred under reflux for 72 h at 50 °C. At the end of this period, the reaction mixture was cooled to room temperature and poured into ethanol. The obtained polymer was collected by filtration and washed several times with ethanol. The polymer was then filtered and dried in a vacuum oven at room temperature for 24 h.

FT-IR (ATR, cm^−1^): 3200–3000 (Ar), 2975(–CH_3_), 2945(–CH_3_), 1320 and 1290 (O=S=O), 1240 (C–O–C), 1175 and 1150 (O=S=O) and 1015 (Ar).

^1^H-NMR (CDCl_3_, ppm): *δ* = 7.9–6.9 (m, Ar), 4.5 (s, CH_2_–Cl), 1.7 (s, CH_3_).

### 2.4. Azidation of chloromethylated polysulfone (PSU–CH_2_–N_3_)

The obtained PSU***–***CH_2_ ***–***Cl (0.1 mmol, 3.0 g) was dissolved in 50 mL of DMF in a round-bottom flask. Sodium azide (1.5 mmol, 0.1 g) was added to the solution and the reaction temperature was increased to 60 °C. After stirring for 24 h, the reaction mixture was concentrated and precipitated into technical ethanol. The product (PSU***–***CH_2_ ***–***N_3_) was dried in a vacuum oven for 24 h at room temperature, yielding a white amorphous solid.

FT-IR (ATR, cm^−1^): 3200–3000 (Ar), 3060 (C–H), 2120 (–N_3_), 1320 and 1290 (O=S=O), 1240 (C–O–C), 1175 and 1150 (O=S=O) and 1015 (Ar).

^1^H-NMR (CDCl_3_, ppm): *δ* = 7.9–6.9 (m, Ar), 4.4 (s, CH_2_–N_3_), 1.7 (s, CH_3_).

### 2.5. Synthesis of polysulfone containing pendant benzoxazine units (PSU–CH_2_–Bz) via CuAAC click chemistry

To a Schlenk tube equipped with a magnetic stirring bar, PSU***–***CH_2_ ***–***N_3_ (0.20 g, 26,000 g/mol, 1.0 equiv), P–Pa (0.182 g, 550 g/mol, 3.3 equiv), ligand (2,2′-dipyridyl, 0.33 mmol), catalyst (CuBr, 0.33 mmol) were dissolved in 10 mL of DMF. The tube was degassed by three freeze–pump–thaw cycles, left under vacuum, and placed in a thermostated oil bath. After stirring at 60 °C for 24 h, the mixture was diluted with THF and then passed through a column of neutral alumina to remove metal salt. The reaction mixture was concentrated and precipitated into methanol. The ﬁnal product was washed with methanol and dried for 24 h in a vacuum oven at 25 °C, yielding a white amorphous solid.

### 2.6. Characterization

^1^H-NMR spectra of 5–10% (w/w) solutions of the intermediates and final polymers in CDCl_3_ and DMSO with Si(CH_3_)_4_ as an internal standard were recorded at room temperature at 500 MHz on a Bruker DPX 500 spectrometer. FT-IR spectra were recorded on PerkinElmer FT-IR Spectrum One spectrometer with an ATR Accessory (ZnSe, Pike Miracle Accessory) and cadmium telluride (MCT) detector. Resolution was 4 cm^−1^ and 24 scans with 0.2 cm/s scan speed. DSC was performed on a TA Q2000. Thermal gravimetric analysis (TGA) was performed on Perkin-Elmer Diamond TA/TGA with a heating rate of 10 °C min under nitrogen flow. Molecular weights and polydispersities of the polymers were measured by gel permeation chromatography (GPC) employing an Agilent 1100 instrument equipped with a differential refractometer by using tetrahydrofuran as the eluent at a ﬂow rate of 0.5 ml min^−1^ at 30 °C. Molecular weights were determined using polystyrene standards.

## 3. Results and discussions

**Scheme 1.**
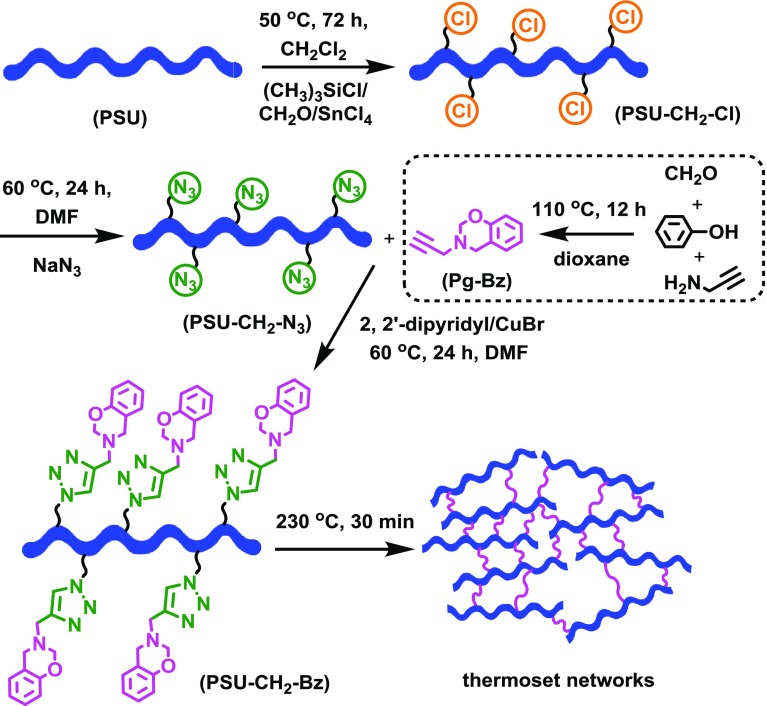
Synthetic route for the PSU-CH_2_-Bz and its components (PSU-CH_2_-Cl, PSU-CH_2_-N_3_ and Pg-Bz).

In practical applications, low molecular weight benzoxazine monomers were heated to elevated temperatures in order to enable the ring-opening reactions of oxazine without using any catalyst. However, the obtained thermoset networks suffered from the brittleness, which was the primary issue when exploring thin film applications. To overcome this problem, new strategies involving a combination of various thermoplastic polymers with benzoxazine units as curable precursors was developed. Generally, thermally curable benzoxazine moieties were located either main- or side- or end-chain of polymeric structures generating a processable and cross-linkable thermoplastic having flexibility and high cross-linking density after cure. In our case, the overall procedure involving the synthesis of chloromethylation and azidation of polysulfone and its click reaction with 3,4-dihydro-3-(prop-2-ynyl)-2H-benzooxazine monomer (Pg***–***Bz) to form self-curable polysulfone (PSU***–***CH_2_ ***–***Bz) was shown in Scheme 1. Firstly, chloromethylated polysulfone (PSU***–***CH_2_ ***–***Cl) was prepared with low degree of substitution (15%) according to modified halomethylation procedure.[53]

The clickable alkyne-functional benzoxazine Pg***–***Bz, and azide-functional polysulfone (PSU***–***CH_2_ ***–***N_3_) were formed by reaction of paraformaldehyde, phenol and propargylamine and nucleophilic substitution reaction of the chlorine atoms of PSU***–***CH_2_ ***–***Cl with excess sodium azide, respectively. Thereafter, the successful CuAAC click reaction between PSU***–***CH_2_ ***–***N_3_ and Pg***–***Bz monomer led to self-curable polysulfone possessing pendant benzoxazine groups, PSU***–***CH_2_ ***–***Bz. Finally, the thermoset networks, where the ring-opening of benzoxazine units of resulting polymer could convert partial linear structures to the network structures, were obtained by simple heating without curing agents.

**Figure 1.**
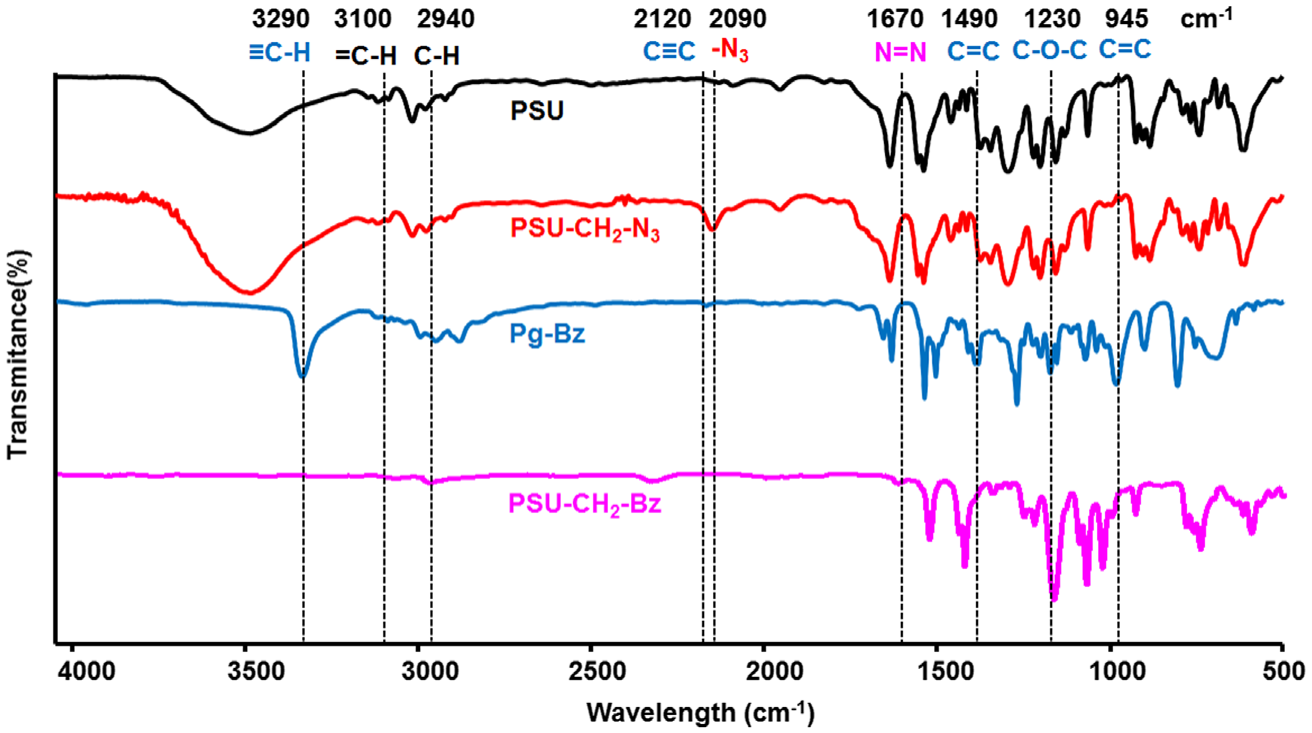
FT-IR spectra of PSU, PSU–CH_2_–N_3_, Pg***–***Bz, and PSU–CH_2_–Bz compounds.

The synthesis of PSU***–***CH_2_ ***–***Bz was monitored by FT-IR analysis following characteristic azide, alkyne and aromatic bands of starting compounds (PSU***–***CH_2_ ***–***N_3_ and Pg***–***Bz) (Figure 1). The FT-IR spectrum of Pg***–***Bz showed ≡C–H and C≡C stretching vibrations at 3290 and 2120 cm^−1^, whereas the C=C stretching vibration derived from the 1,2,4- substitution of the benzene ring appeared at 1490 cm^−1^ and the asymmetric stretching of C–O–C at 1230 cm^−1^. In the case of PSU***–***CH_2_ ***–***N_3_, azidation of chloromethylated polysulfone was followed by appearance of a strong azide peak arising at 2090 cm^−1^ indicating the presence of azide groups. After the CuAAC ‘click’ reaction, the disappearance of the azide and alkyne peaks and appearance of new peaks from the benzene ring of the Pg***–***Bz in the in the FT-IR spectrum of PSU–CH_2_–Bz confirmed the structure of desired polysulfone bearing pendant benzoxazine units. Also, the emergence of the N=N peak of triazole ring at 1670 cm^−1^ and the disappearance of ≡C–H peak at 3290 cm^−1^ indicated the successful CuAAC ‘click’ reaction.

**Figure 2.**
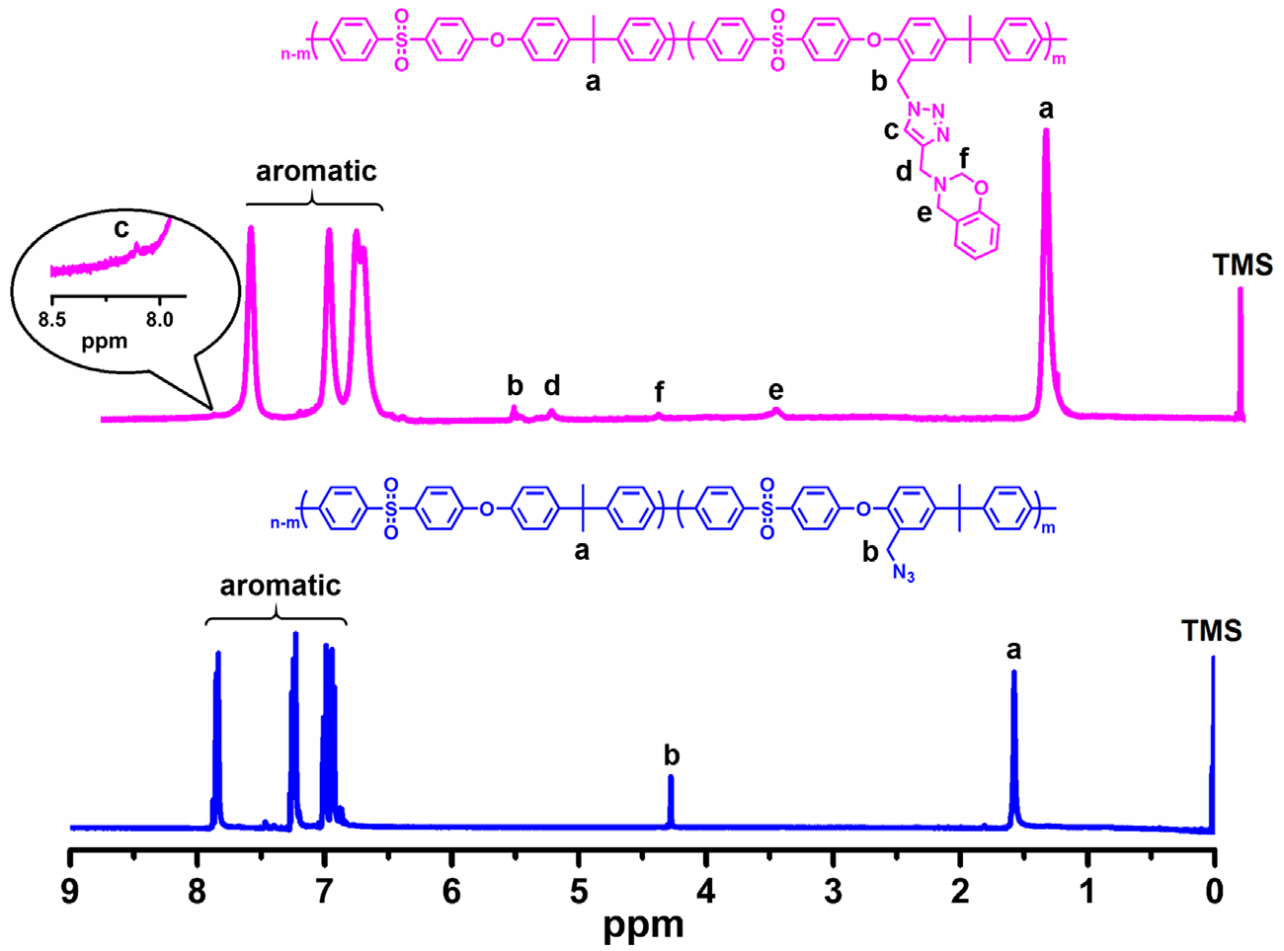
^1^H NMR spectra of PSU–CH_2_–N_3_ and PSU–CH_2_–Bz compounds.

The structures of the PSU–CH_2_–Bz was further confirmed by ^1^H-NMR analysis as shown in Figure 2. The proportion of chloromethylated repeating units on PSU chains (degree of chloromethylation) was determined by the ratio of the integration values of the signals corresponding to the peak at 4.5 to the peak at 7.8 belonging to meta protons on the benzene ring near to the sulfonyl group. The degree of chloromethylation was determined to be 15%. After azidation reaction, the signals corresponding to methylene protons shifted from 4.5 to 4.4 ppm indicating successful azidation with 100% conversion (Figure S1). Successful click reaction was also proven by ^1^H-NMR spectra of the PSU–CH_2_–Bz that exhibited complete removal of the signal 4.4 ppm shifting to the position at 5.3 ppm. The attachments of the benzoxazine units were further proved by the appearance of the characteristic peaks at 3.6 and 4.9 ppm, corresponding to protons in the methylene bridge of the oxazine (Figure 2). Moreover, the structure of PSU–CH_2_–Bz was also confirmed by ^13^C-NMR spectroscopy. The disappearance of the propargyl group signals at 73.1 and 78.2 ppm, and the appearance of triazole signals of PSU–CH_2_–Bz at 121.4 and 144.6 ppm indicated the successful CuAAC click reaction (Figure S2).[54]

The molecular weight characteristics of the pure PSU and modified PSU were also monitored by GPC analysis (Table 1). The molecular weight and molecular weight distribution of the modified PSU were slightly increased after the synthesis steps and no degradation products with small molecular weights were detected during the whole process.

**Table 1.** Molecular weight characteristics of pure PSU and modified PSU derivatives.

Sample	*M*_n_^a^(g/mol)	*M*_w_/*M*_n_^a^
PSU	28.400	3.07
PSU–CH_2_–Cl	30.000	2.96
PSU–CH_2_–N_3_	32.000	3.31
PSU–CH_2_–Bz	34.000	2.75

^a^ Number average of molecular weights and molecular weight distributions were determined by GPC equipment based on polystyrene standards.

**Figure 3.**
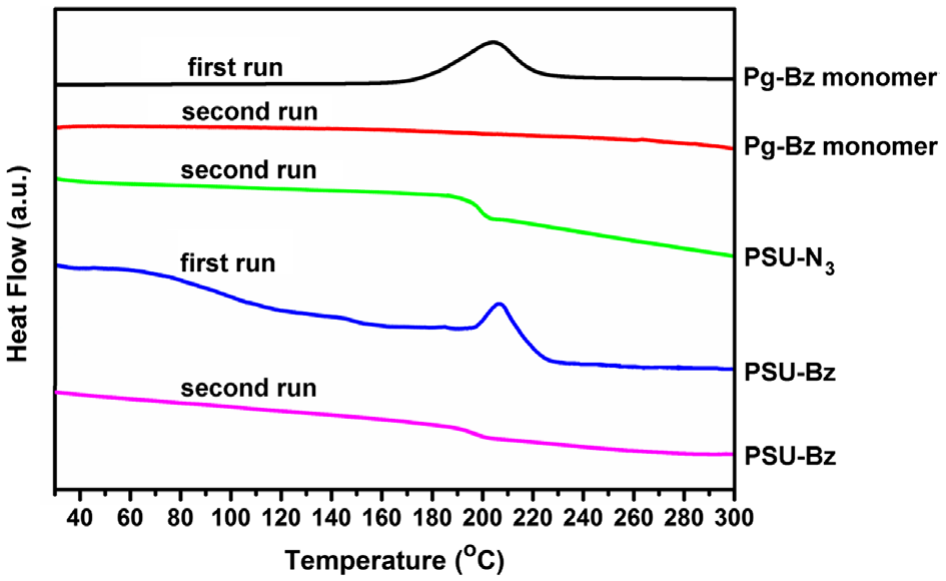
DSC thermograms of Pg-Bz, PSU-CH2-N3 and PSU-CH2-Bz compounds.

The curing behavior of Pg***–***Bz and PSU–CH_2_–Bz was investigated by DSC analysis and obtained thermograms were presented in Figure 3. The PSU–CH_2_–N_3_ exhibited a glass transition temperature at 189 °C due to amorphous nature of polysulfone. The ring-opening reaction of Pg***–***Bz monomer had an exotherm with a maximum at 206 °C. The amount of the exotherm reaction heat of Pg***–***Bz was calculated 943 J/g and it disappeared completely after the 230 °C cure. In the second run there was no thermal transition indicating the complete ring-opening reactions and the formation of polybenzoxazine network. In the case of PSU–CH_2_–Bz, the sample displayed an exotherm at 206 °C, which could be assigned to the ring-opening reaction of benzoxazine moieties. In the second run, there was no exotherm as the result of the formation of thermoset and completion of ring opening reaction. Due to the linear polymer chains, the sample still had a broad *T*_g_ at 196 °C, which increased by the addition of hard benzoxazine units and triazole linkages.

**Figure 4.**
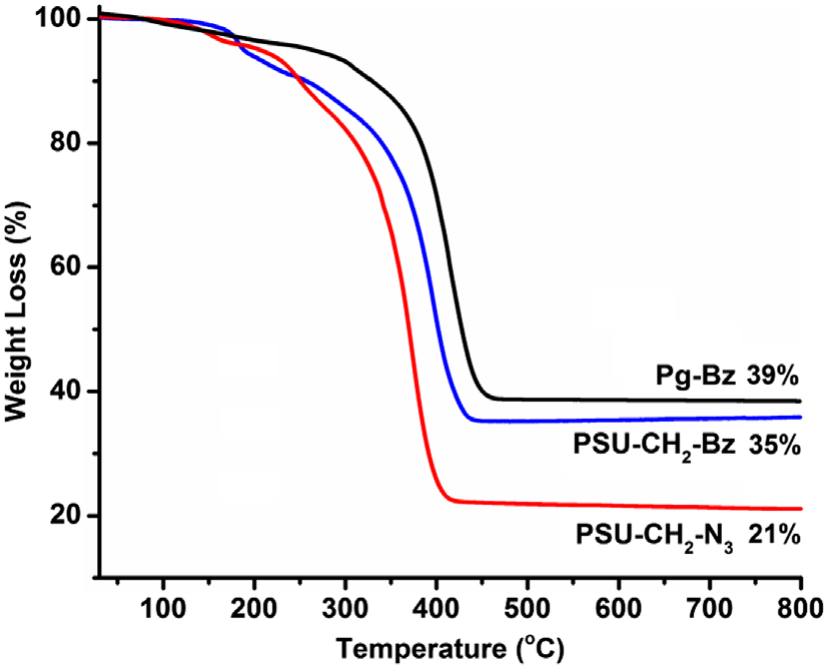
TGA thermograms of Pg-Bz, PSU-CH_2_-N_3_ and PSU-CH_2_-Bz compounds cured at 230 °C for 30 min.

TGA thermograms of Pg***–***Bz, PSU–CH_2_–N_3_ and PSU–CH_2_–Bz samples were shown in Figure 4. The thermograms involve preheating cycle in which the samples were heated up to 230 °C, allowed to stay at this temperature for 30 min and then cooled down to ambient temperature followed by heating to 800 °C. The thermal degradation of cured Pg***–***Bz started at 270 °C and completed at 450 °C, which was well above that of polybenzoxazine. In case of PSU–CH_2_–N_3_, the main degradation occurred between 300 and 470 °C with a single step. When polysulfone and benzoxazine groups were combined in the network, thermal stability was increased with onset degradation temperature was 440 °C. Compared to the PSU–CH_2_–N_3_, the char yield of cured PSU–CH_2_–Bz was increased with the addition of benzoxazine units. At high temperatures, the cured PSU–CH_2_–Bz sample exhibited greater thermal stability, which might be due to the hydrogen bonding interactions between phenolic groups of benzoxazine and sulfoxide groups on the main chain.[10] Similar results were also obtained from previously reported study, in which thermosets prepared from benzoxazine end-functional polysulfone.

## 4. Conclusion

In conclusion, a self-curable polysulfone bearing pendant benzoxazine units was successfully prepared by CuAAC click chemistry. By simple heating of this polymer allowed to form thermoset network containing polysulfone and benzoxazine groups in the structure. The benzoxazine moieties contribute to enhance the thermal properties of the polysulfone networks. This work may be useful for the obtainment of new polymeric networks that can be used in membrane science as ultrafiltration membranes or coating materials.

## Supplemental data

The Supplemental data for this article is available online at http://dx.doi.org/10.1080/15685551.2016.1257379.

## Disclosure statement

No potential conflict of interest was reported by the authors.

## Supplementary Material

TDMP_1257379_Supplemental_Material.zip
