# Supplementary material for: Synthesis of self-curable polysulfone containing pendant benzoxazine units via CuAAC click chemistry
Source: Des Monomers Polym. 2016 Nov 21;20(1):293–9. doi: 10.1080/15685551.2016.1257379 (PMC5812181; doi:10.1080/15685551.2016.1257379)
Supplement: TDMP_1257379_Supplemental_Material.zip [file TDMP_A_1257379_SM0588.zip › TDMP_1257379_Supplemental_Material.docx]

**Supporting Information**

**Synthesis of Self-Curable Polysulfone Containing Pendant Benzoxazine Units via CuAAC Click Chemistry**

**Cemil Dizman^1*^, Cagatay Altinkok ^2^, Mehmet Atilla Tasdelen^2*^**

*^1^Institute of Chemical Technology, TUBITAK Marmara Research Center, Gebze, Kocaeli 41470, Turkey*

*^2^Department of Polymer Engineering, Faculty of Engineering, Yalova University, 77100
 Yalova, Turkey*

*Corresponding authors

E-mail: cemil.dizman[@](mailto:yusuf@itu.edu.tr)tubitak.gov.tr, Fax: +90 262 641 23 09; Phone: +90 262 677 38 42

E-mail: tasdelen[@](mailto:yusuf@itu.edu.tr)yalova.edu.tr, Fax: +90 226 815 54 01, Phone: +90 226 815 54 12


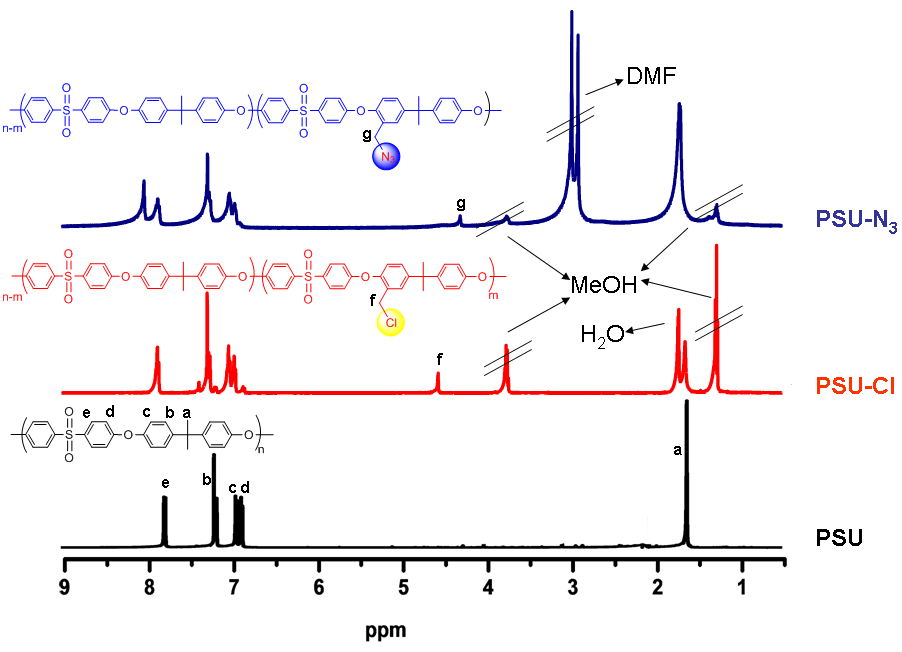


**Figure S1.** ^1^H-NMR spectra of PSU, PSU-CH_2_-Cl and PSU-CH_2_-N_3_ compounds.


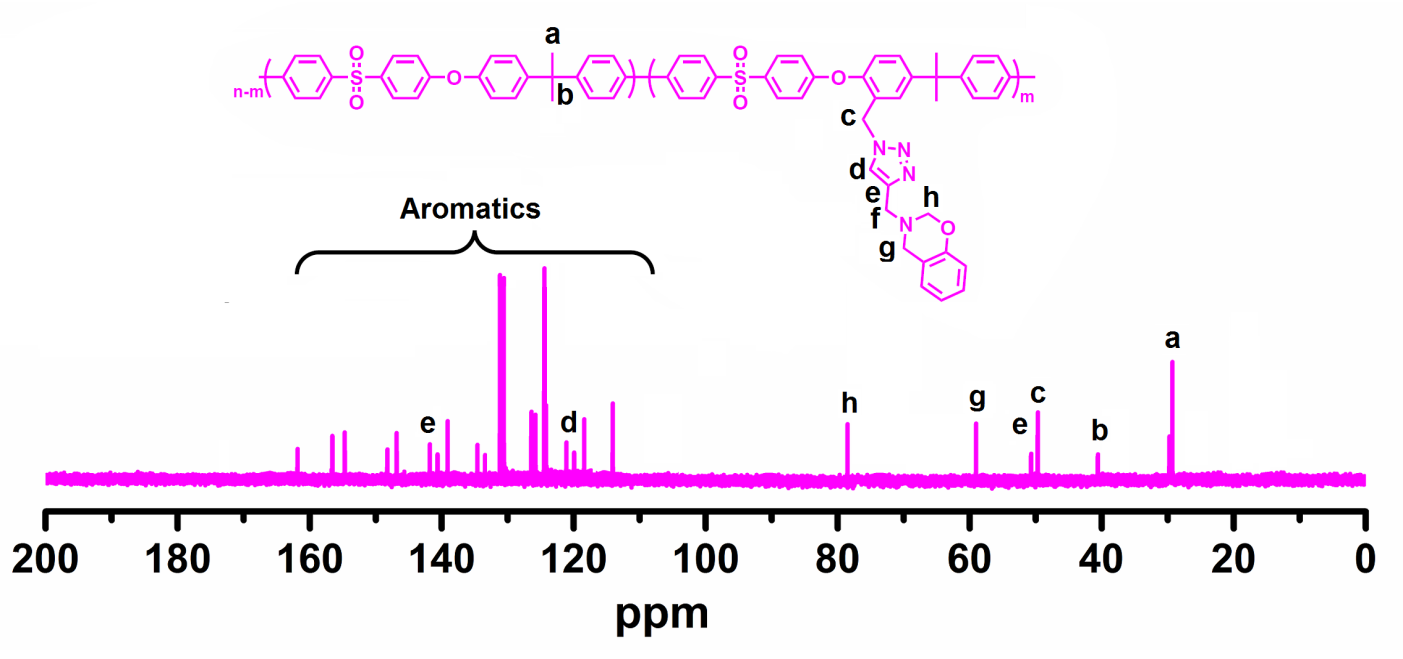


**Figure S2.** ^13^C-NMR spectrum of PSU-CH_2_-Bz compound.
